# The impact of COVID-19 on the doctor-patient relationship in China

**DOI:** 10.3389/fpubh.2022.907009

**Published:** 2022-08-01

**Authors:** Bo Xu

**Affiliations:** Medical Humanity and Information Management College, Hunan University of Medicine, Huaihua, China

**Keywords:** doctor-patient relationship, doctor-patient conflict, communication, COVID-19, China

## Abstract

A strong doctor-patient relationship (DPR) is crucial to the effectiveness of treatment. It is imperative to maintain a good DPR during treatment. During 2019, Coronavirus Disease 2019 (COVID-19) brought new challenges to already difficult doctor-patient relationships. This paper summarized the current state of the DPR, compared the changes between China and other countries after the outbreak of COVID-19, and listed the solutions proposed by various countries. Finally, the author suggested some solutions in order to improve the DPR according to China's own circumstances.

## Introduction

A harmonious doctor-patient relationship (DPR) is the basis for a successful therapeutic outcome. In the relationship between physicians and patients in Chinese society today, contradiction plays a prominent role. Having gained extensive experience from their friends, relatives, and neighbors, the general population has a better understanding of DPR. The public is not satisfied with the DPR in multiple countries (1–3). Reasons for this included unreasonable time allocation for patients, unclear explanations regarding prescriptions, induction of misinformation on the Internet and social status discrimination against patients. However, the conflict cannot simply be attributed to one party. An employee's emotional orientation at work determines job satisfaction (4). According to doctors, balancing professional and personal life is difficult, and work-family conflicts put doctors and nurses under a great deal of stress (5, 6). Their enormous work load made it difficult for them to maintain a positive attitude. Worse, they feared that patients would become aggressive, making it difficult to ensure their safety.

Communication is at the heart of medicine. Zolnierek found that if physicians were good communicators, the odds of having adherent patients were doubled (7). Patients and physicians are unable to communicate effectively due to communication barriers and language ambiguity (8, 9). Often, young children, the elderly, and mental patients are unable to adequately describe their symptoms and have a poor attitude toward doctors. Communication skills and attitude are especially important in these situations (10). Listening, sincerity, trust, and appropriate empathy all contribute to the effective communication (11). Good doctor-patient communication will make patients open their hearts, trust doctors, and prefer cooperation, so that doctors could understand patients' conditions and make effective diagnosis in time (12, 13). Higher service quality at the hospital leads to higher customer trust, a better customer experience, and a stronger DPR (14). Communicating improves patient satisfaction and decreases lawsuits. Patient satisfaction will reduce complaints to the hospital, thus reducing the pressure to the doctors. Yet, the outbreak of Coronavirus Disease 2019 (COVID-19), which brought new challenges to the DPR, exacerbated an already strained relationship. Several countries, including China, have investigated the DPR in their own countries to determine how the COVID-19 has affected it, resolve the original contradiction and find a new approach to the DPR.

## Influence of COVID-19 on global DPR

COVID-19 has disrupted communication and worsened the DPR. COVID-19 transmits mainly through droplets, with a long incubation period (14–28 days) (15, 16). As a result of COVID-19, individuals may experience respiratory symptoms, fever, shortness of breath, cough, dyspnea, and viral pneumonia, and in severe cases, acute respiratory syndrome, heart failure, and even death (17). The COVID-19 epidemic was at its peak in its early stages, before effective therapeutic vaccines were available. Hospitals were once full due to the large number of infected individuals. Those suffering from COVID-19 in Mexico were kept isolated in hospitals, not only from other patients but also from all members of the medical staff, their families, and friends (18). Wearing defensive clothing and masks prevented patients from seeing the doctors' facial expressions, resulting in a reduction of physical contact and a limitation of communication. Most people feel helpless and lonely during an extended quarantine (19).

Chronic patients (diabetes, hypertension, uremia, etc.) display higher mortality rates of COVID-19, which exacerbates the symptoms of patients with chronic diseases and weakens their immunity. Due to the decrease in immunity, COVID-19 is more likely to be diagnosed in patients undergoing dialysis and kidney transplantation (20). COVID-19 promotes the onset of chronic diseases related complications (21). In order to prevent unnecessary infection in the hospital as well as to decrease hospital gatherings, it is recommended that patients should be isolated at home. As a result of home isolation, chronic patients are denied drugs and treatment, and are unable to communicate with doctors and obtain medical treatment in time. During the COVID-19 pandemic in Brazil, the quality of care received by patients with type 1 diabetes has declined significantly due to isolation at home, and patients on several measures of quality of care have declined significantly (22). Once patients were forced to seek medical treatment, the hospital required them to have negative nucleic acid test results in order to be hospitalized. A long period of early detection of nucleic acids may delay the most effective period of treatment for patients, resulting in several tragedies.

National Health Service (NHS) developed the “Your COVID recovery” website (http://www.yourcovidrecovery.nhs.uk) in response to these issues to provide online support for people who were in home isolation (23). US dermatologists were encouraged to use video conferencing to consult with patients during COVID-19 (24). It can be used to communicate with doctors by showing infected or diseased areas. Video consultation will also be beneficial to young people suffering from type I diabetes who are experiencing disease and emotional stress during the COVID-19 pandemic (25). A second measure taken by the government is to include telemedicine services within the scope of medical insurance reimbursement, and to increase the amount of reimbursement under medical insurance (26). Swiss eHealth services offer effective, scalable, and cost-effective treatments for people who lack access to mental health services (27). However, in practice this type of telemedicine still has limitations, as the quality of remote “physical” consultation is lower than the quality of actual physical examination, and as well, it can easily destroy the quality of doctor-patient interaction for a number of reasons (28). The above solutions can be understood and supported by most rational individuals. Sophia et al. studied patients' satisfaction with empathy and communication of emergency doctors in Arizona by using the Hospital Consumer Assessment of Healthcare Providers and Systems during COVID-19. The results indicated that patients understood doctors in the special circumstances, and their scores for doctors were above the average (29). Because they felt the hard work and efforts of doctors to fight the epidemic. These have helped ease the conflict between doctors and patients during COVID-19 to a certain extent.

## The influence of COVID-19 on DPR in China

In Jinan, Shandong province, China, Zhanming Liang distributed paper questionnaires to doctors and patients at two hospitals at the site of the COVID-19 outbreak. In spite of the fact that the majority of health care workers recognize the importance of patient-centered care, appropriately 20% of them are unaware of the importance of patient counseling (30). Since 2020, DPR has been steadily improving. The Second Xiangya Hospital of Central South University, in collaboration with Texas Tech University, conducted a few interviews with doctors and patients during COVID-19 in order to gain an understanding of the DPR in China. Participants were given the opportunity to complete some questionnaires distributed on several popular social networking sites (QQ, Wechat) used in China. According to the cross-sectional study, patient characteristics such as age, income, occupation, and residence do not significantly affect the results of the Patient-Doctor Relationship Questionnaire (PDRQ) (31). PDRQ scores increased from 34.74 before the epidemic to 37.65 during the epidemic, indicating a positive change in the DPR. Patients have high levels of trust in doctors as indicated by the Wake Forest doctor trust scale. According to their report, the level of medical violence against doctors and other co-workers decreased by about 20% during COVID-19.

A doctor's perspective is then taken into account during the investigation. Zhou et al. conducted a survey of 979 healthcare professionals, including doctors, nurses, and technicians, at COVID-19. Researchers found that professionals with bachelor's and master's degrees or higher had a low level of trust for patients. A DPR survey conducted in Beijing in 2018 found that physicians rated trust more negatively than patients, and their trust scores were lower than their patients due to doctor-patient disputes and inequalities of information (32). Yet, during the COVID-19 pandemic, they were treated with more respect than before. In the epidemic, both verbal and physical violence against themselves and their colleagues declined significantly. In a Pearson correlation analysis, Zhou et al. found that positive media coverage of medical staff, free online consultations, a psychiatric hotline and free treatment for confirmed and suspected COVID-19 patients had a significant positive impact on doctor-patient relationships (33).

**Figure 1:**
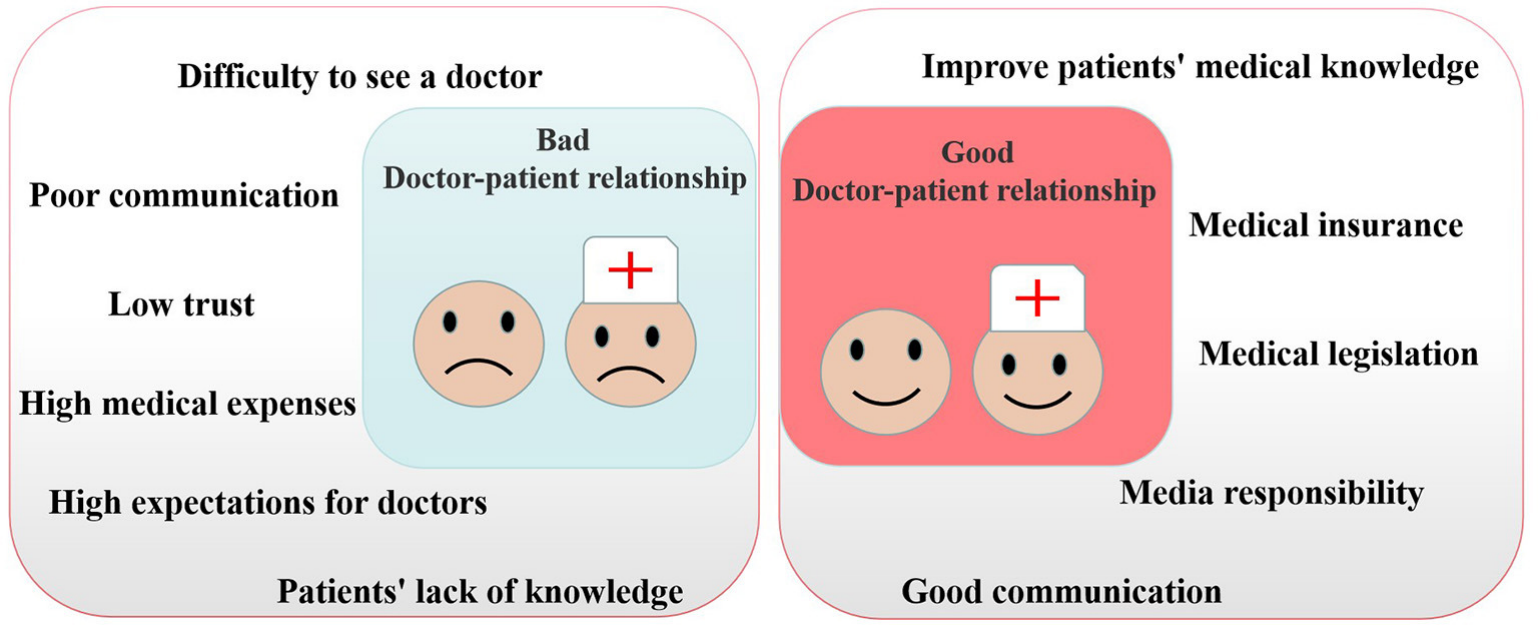
Factors affecting doctor-patient relationship.

From the perspective of patients, the main reasons for the contradiction between doctors and patients were difficulty to see a doctor, high medical expenses, high expectations for doctors, low trust, patients' lack of knowledge and poor communication. As a consequence of these findings, it appears that COVID-19 did not resolve the doctor-patient contradiction, but instead sped up the process of resolving it (34). Cochran's *Q*-test demonstrated important factors of enhancing DPR were the improvement of medical legislation, good communication, patients' basic medical knowledge, media responsibility and medical insurance (Figure 1) (31, 33). Hence, with regard to the conditions in China or in other countries, improving doctor-patient communication, medical technology, and patients' medical knowledge may contribute to improving doctor-patient relationships.

## Discussion

The DPR has its own particularities based on national differences. In the short term, it will be difficult to improve the doctor-patient relationship in China. However, a new round of reforms to the medical system may be able to resolve and prevent doctor-patient conflicts, thereby achieving a more healthy and harmonious care-giving relationship (35). We should consider the following aspects.

The first step to repairing the DPR is to improve their communication. It is helpful if doctors reduce the use of obscure specialized vocabulary during the communication process, and answer the patient's questions in a more accessible manner. It has been shown that non-verbal communication can improve doctor-patient communication, such as eye contact, control of body posture and movement, and tone and speed of speech (36). These can build patients' confidence and establish a positive relationship. Another important point is that treatment-related adverse events are largely related to the cognitive gap between doctors and patients. A study in China by Yu et al. proposed the inclusion of patient reported results in clinical trials and routine clinical practice (37). This approach may reduce doctor-patient conflict resulting from poor prognoses.

Secondly, we should increase the number of doctors and give each patient enough time to visit. Until the outbreak of the epidemic, doctors in the capital cities of provinces were only able to see patients for 3–5 min, and the amount of time individuals were allowed to see a patient was diminished considerably (38). After the outbreak of COVID-19, doctors have become more difficult to interview. A few doctors have taken advantage of social media sites (such as WeChat and QQ) to assist their patients, especially those with chronic diseases or who are pregnant. This could expedite the treatment of more patients in a short amount of time. In spite of this, doctors who do so are still in the minority, since they should be compensated appropriately for their work. Hospitals may therefore create their own official online fee-based consultation service. As has been done by the U.S. above, the government may be able to include the costs in the scope of medical insurance reimbursement.

Thirdly, we should improve the job satisfaction of doctors. Doctors' work attitude and enthusiasm are affected by their job satisfaction (39). In 2013, a survey found that doctors in Hubei Province, China, were not satisfied with their work (40). 64.8% of doctors in Shanghai's tertiary public hospitals were dissatisfied with their careers in 2019 (41). Their dissatisfaction was based on the doctor's title, department, work hours, and requirements as well as the stress of their life and work schedules. By restructuring the existing medical system, reducing the burden on doctors, and improving the treatment of physicians, we can indirectly improve the DPR as well (42).

Fourth, ensure the training of medical students is focused on doctor-patient communication ability. Practicing nurses revealed that the higher their self-efficacy level, the better their ability to communicate with patients. The trainee nurses who have undergone self-efficacy training have more effective nurse-patient communication skills than those who have not received training (43, 44). For this reason, during the education of medical students and nurses, the training of doctor-patient interaction should be emphasized, in order for them to function calmly and efficiently in the future. In 2019, the Indian Medical College introduced competency-based medical education and the use of skills labs and simulation guides to improve the daily learning experience of students by simulating the needs and experiences of real patients, assisting them in the transition to seeing real patients (45). Designing a course should focus on enhancing the creativity and extroversion of medical students, and maintaining a positive attitude will facilitate communication between the doctor and the patient (46).

Last but not least, rectifying the misleading reports of the media. At the start of the COVID-19 outbreak, the irresponsible media attempted to link this outbreak to other diseases that share similar symptoms, such as Chronic Fatigue Syndrome/Myalgia Encephalomyelitis (CFS/ME) or Post-Viral Fatigue, which made it difficult for doctors to diagnose the illness and made the public misinformed (23, 47). In order to prevent patients from being deceived by inaccurate information and having to bear serious consequences, accurate medical science knowledge should be spread by official media. The media should report more positive stories about the DPR. If the tragedies occurred, they should obtain the truth from the facts rather than making up facts out of context for the purpose of blogging.

It is a long and difficult process to improve the DPR, but finding ways to benefit physicians and patients is still essential. It would not be appropriate to copy the methods of other countries, but instead, we should start from our own situation and find a method that is appropriate to our needs.

## Author contributions

BX conceptualized the idea for this manuscript and drafted and finalized the manuscript.

## Conflict of interest

The author declares that the research was conducted in the absence of any commercial or financial relationships that could be construed as a potential conflict of interest.

## Publisher's note

All claims expressed in this article are solely those of the authors and do not necessarily represent those of their affiliated organizations, or those of the publisher, the editors and the reviewers. Any product that may be evaluated in this article, or claim that may be made by its manufacturer, is not guaranteed or endorsed by the publisher.
